# Retrospective Evaluation of Dystocia in Miniature Equids: 78 Cases (2002–2023)

**DOI:** 10.1111/vec.70014

**Published:** 2025-08-21

**Authors:** Ari Merari, Langdon Fielding

**Affiliations:** ^1^ Loomis Basin Equine Medical Center Penryn California USA

**Keywords:** dystocia, foal, mare, miniature donkey, miniature horse

## Abstract

**Objective:**

To describe patient characteristics, laboratory data, fetal orientation, methods of correction, survival, and treatment in miniature equids with dystocia.

**Design:**

Retrospective study conducted from January 2002 to June 2023.

**Setting:**

Equine referral hospital and field service.

**Animals:**

Seventy animals with a total of 78 instances of dystocia.

**Interventions:**

None.

**Measurements and Main Results:**

Recorded variables included signalment, clinicopathologic data, fetal presentation, correction method, survival to discharge, and complications. The survival of miniature equid mares was 94% (73/78), while survival of foals was 17% (13/78). The most common causes of dystocia were abnormal limb presentation in 27% (21/78), neck flexion in 23% (18/78), and caudal presentation with bilateral hip flexion in 17% (13/78). The correction methods used were controlled vaginal delivery in 45% (35/78), assisted vaginal delivery in 35% (27/78), cesarean delivery in 17% (13/78), and fetotomy in 4% (3/78). Complications included retained fetal membranes in 19% (15/78), metritis in 8% (6/78), obturator nerve paralysis in 5% (4/78), and hyperlipidemia in 5% (4/78).

**Conclusions:**

Miniature equid mares appear likely to survive dystocia. The condition can be resolved by controlled or assisted vaginal delivery in the majority of cases. Continued research and training are needed to improve survival in foals.

**Abbreviations**
AVD: assisted vaginal delivery
CRI: continuous rate infusion
CVD: controlled vaginal delivery
RFM: retained fetal membranes

## 1 Introduction

Dystocia, characterized by a prolonged or abnormal second stage of parturition, is an emergency in equine practice, with equine births having a reported prevalence of dystocia between 2% and 13% [1]. In horses, stage 2 parturition begins with the rupture of the chorioallantoic membrane. This stage is short and lasts, on average, 20–30 min [2]. The longer it takes to resolve the dystocia (>30 min), the greater the incidence of complications and mortality of the foal and mare [2, 3, 4]. Proactive and efficient intervention is critical for resolution of dystocia and increases the likelihood of a positive outcome. The causes of dystocia are usually related to abnormalities in the foal's presentation, positioning, or size, but occasionally dystocia results from factors associated with the mare [5]. The ideal outcome of dystocia is a live foal and mare with minimal or manageable complications.

Common complications of dystocia in foals include asphyxia, hypoxia, associated hypoxic ischemic encephalopathy, and failure of passive transfer. Survival of the foal depends largely on the duration of dystocia [6]. In one study, all foals delivered 90 or more minutes after the start of stage 2 labor were deceased [7]. Foals born alive or surviving to hospital discharge with stage 2 labor longer than 90 min have been reported, but this is not common [4, 8]. The most frequently reported mare‐associated complications include retained fetal membranes (RFM), metritis, laminitis, trauma to the urogenital tract, uterine artery rupture or hemorrhage, uterine prolapse, and peripheral neuropathy [9]. Mortality from postpartum complications has been reported to range from 1.5% to 12% [7, 9, 10, 11].

Techniques to resolve dystocia have been described elsewhere and include assisted vaginal delivery (AVD) with the mare standing, controlled vaginal delivery (CVD) with the mare anesthetized in dorsal recumbency, cesarean delivery, and fetotomy [4]. Alternatively, if dystocia cannot be corrected and there is no option for cesarean delivery or fetotomy (assuming the foal is deceased), euthanasia can be performed [6, 12].

Despite significant literature describing equid dystocia in full‐sized horses, data and reports in miniature breeds are lacking. With the popularity of miniature breeds, an understanding of the factors associated with dystocia is important to improve veterinarian intervention and animal care. The objective of the current study was to describe the survival, complications, in utero positioning, correction methods, clinical examination findings, signalment, laboratory data, and postdystocia treatment in miniature equids.

## 2 Materials and Methods

Medical records from the Loomis Basin Equine Medical Center from January 1, 2002, through June 30, 2023, were searched. The keyword “dystocia” was used to search the records, which were additionally filtered to include only medical records of miniature equids. Variables that were recorded from the medical records included signalment, physical examination parameters, laboratory data, position of the fetus, and the method of correction of the dystocia.

Where appropriate, variables were evaluated using the Kolmogorov–Smirnov test for normality. Results were reported as mean ± SD or median (range) depending on the distribution. Continuous variables were compared using an unpaired *t*‐test or a Mann–Whitney test depending on the normality of the data. A commercial software program was used for the analysis^a^.

## 3 Results

**FIGURE 1:**
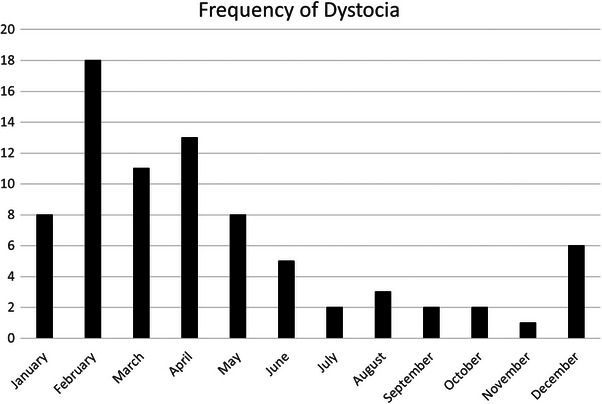
Frequency of 78 instances of dystocia in 70 miniature equids from January 2002 to June 2023 by month of the year.

**FIGURE 2:**
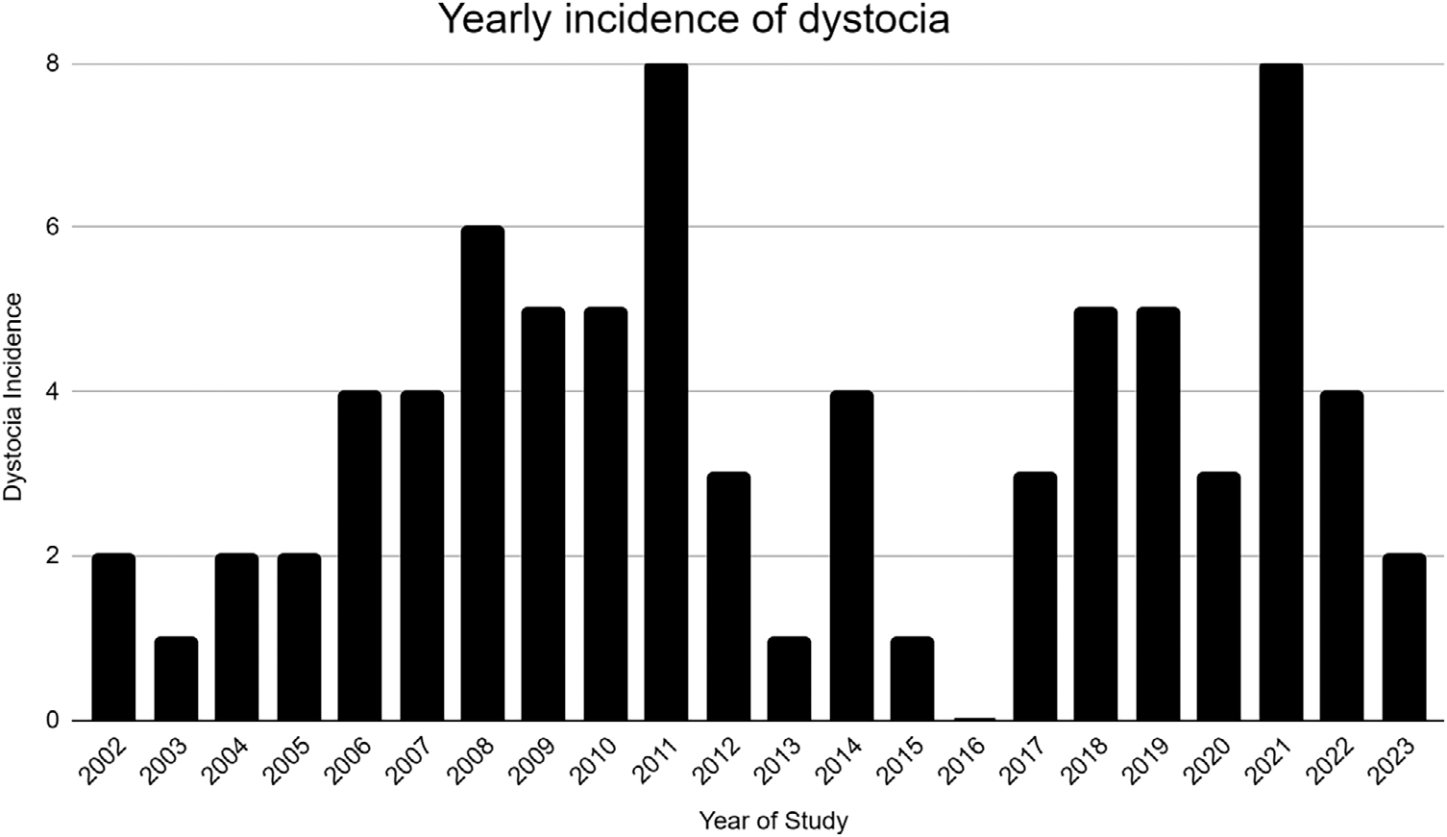
Yearly distribution of 78 instances of dystocia in 70 miniature equids from January 2002 to June 2023.

The current study comprised 78 instances of dystocia in 70 mares. Of these 70 mares, 45 miniature horses and 25 miniature donkeys were included. In 32 instances of dystocia, 41% (13/32) of mares were recorded as primiparous and 59% (19/32) as multiparous. Fourteen percent (11/78) of dystocia cases were ambulatory, 64% (50/78) were in‐hospital only, and 25% (17/78) were attended first in the field and then referred for additional intervention or hospital care. For the 48 cases transported to the hospital for which reliable information was available, a distance of 20 (6–74) km was traveled to reach the hospital. Signalment, physical examination, and laboratory data are presented in Table 1. The monthly distribution of dystocias is shown in Figure 1 and yearly distribution across the study period in Figure 2. Differences in selected variables between miniature horses and miniature donkeys are shown in Table 2.

**TABLE 1:** Signalment, physical examination findings, and laboratory values (reported as mean +/‐ SD or median [range]) for miniature equids presenting for 78 instances of dystocia.

Variable	Number of animals	Values
Age (years)	59	7 (2–22)
Temperature (°C)	13	36.3 (32.8–39.1)
Heart rate (/min)	19	60 (40–160)
Respiratory rate (/min)	15	28 ± 13
White blood cells (10^9^/L) (10^3^/µL)	27	8.4 ± 3.4
Neutrophils (10^9^/L) (10^3^/µL)	21	5.9 ± 3.1
Lymphocytes (10^9^/L) (10^3^/µL)	27	2.1 ± 1.1
Platelets (10^9^/L) (10^3^/µL)	27	178 (32–603)
Packed cell volume (%)	29	36 ± 7
Total protein (g/L) (g/dL)	31	0.68 ± 0.12 (6.8 ± 1.2)
Fibrinogen (µmol/L) (mg/dL)	15	11.5 ± 5.3 (390 ± 180)
Lactate (mmol/L) (mg/dL)	36	4.0 (0.4–14.7), 36 (3.6–132.4)
Bicarbonate (mmol/L) (mEq/L)	30	22.9 ± 4.4
Ionized calcium (mmol/L) (mg/dL)	18	1.4 ± 0.2 (5.6 ± 0.8)
Total calcium (mmol/L) (mg/dL)	26	2.6 ± 0.4 (10.5 ± 1.5)
Blood urea nitrogen (mmol/L) (mg/dL)	34	7.1 ± 2.1 (20 ± 6)
Creatinine (mmol/L) (mg/dL)	33	97.2 (44.2–518.0), 1.1 (0.5–5.86)
Phosphorus (mmol/L) (mg/dL)	5	1.1 ± 0.3 (3.5 ± 0.9)
Albumin (g/L) (g/dL)	26	29 ± 4 (2.9 ± 0.4)
Globulin (g/L) (g/dL)	25	39 ± 7 (3.9 ± 0.7)
Glucose (mmol/L) (mg/dL)	34	10.4 (4.2–29.0), 188 (75–523)
AST (units/L) (U/L)	23	389 (235–1099)
GGT (units/L) (U/L)	26	30 ± 6
Total bilirubin (µmol/L) (mg/dL)	23	18.8 ± 29.1 (1.1 ± 1.7)
LDH (units/L) (U/L)	10	664.5 (375–2719)
CK (units/L) (U/L)	20	605 (151–6581)
Sodium (mmol/L) (mEq/L)	44	141 ± 6
Potassium (mmol/L) (mEq/L)	44	4.0 ± 0.7
Chloride (mmol/L) (mEq/L)	32	103 ± 6

Abbreviations: AST, aspartate aminotransferase; CK, creatine kinase; GGT, gamma‐glutamyltransferase; LDH, lactate dehydrogenase.

**TABLE 2:** Difference in selected variables between miniature horses and miniature donkeys with dystocia.

Variable	Horse	Number of animals	Donkey	Number of animals	*p*‐value
Age (years)	8 ± 4	39	7 ± 5	20	0.24
Temperature (°C)	37 ± 1.5	9	35 ± 2.0	4	0.07
Heart rate (/min)	60 ± 16	14	82 ± 36	7	0.06
Lactate (mmol/L) (mg/dL)	6.3 ± 4.0 (56.8 ± 36.0)	20	3.1 ± 2.2 (27.9 ± 19.8)	16	0.01
PCV (%)	34 ± 7	20	38 ± 6	9	0.15
Total protein (g/L) (g/dL)	65 ± 1 (6.5 ± 1.0)	21	74 ± 1.3 (7.4 ± 1.3)	10	0.05

The overall survival (discharge from the hospital or survival at end of the ambulatory visit) for mares was 94% (73/78). Those that did not survive were all euthanized, for reasons including uterine rupture, severe postoperative ileus, and inability to correct dystocia using CVD and no option to pursue cesarean delivery.

The duration of dystocia was frequently recorded in the medical record as “unknown” or not recorded at all. However, there were comments about the duration of dystocia in 25 cases. In 48% (12/25) of cases, the duration was recorded as <1 h. In 32% (8/25) of cases, the duration was 1–6 h, and in 20% (5/25) of cases, the duration was >6 h.

The overall survival (discharge from the hospital or survival at the end of the ambulatory visit) for foals was 17% (13/78). Twenty‐one percent (16/78) of foals were born alive, and 81% (13/16) of those survived. Of the foals that did not survive, one was euthanized due to marked prematurity and lack of response to resuscitative efforts. A second foal died within 1 h after birth, but additional information about resuscitative efforts or the cause of death was not recorded. A third foal died of an undetermined cause at 2 days of age. Necropsies were not performed.

A wide variety of complications was recorded for mares, the most common of which were RFM in 19% (15/78), metritis in 8% (6/78), obturator nerve paralysis in 5% (4/78), and hyperlipidemia in 5% (4/78). Of the 15 cases of RFM, 47% (7/15) were mares and 53% (8/15) were jennies. Of the 15 cases of RFM, three mares developed metritis, and only one had documented systemic inflammatory response syndrome. No cases of laminitis were reported in any of the mares with RFM. The longest recorded duration of RFM was 48 h. In general, our practice defines RFM as retention beyond 3 h post‐foaling.

Mares that developed obturator nerve paralysis responded to conservative treatment with hobbles or intermittent slinging as needed. Only one mare required continuous slinging in the hospital for 24 h. All of the mares with obturator nerve paralysis had CVD or AVD, and none of the mares that developed obturator nerve paralysis were recorded to have presented in recumbency in the trailer.

Four mares that were documented to have hyperlipidemia either had a previous history of the condition, a history or presenting complaint of hyporexia or anorexia, or significant illness as comorbidities. Of these mares, three were reported to be hyporexic. One of the three hyporexic mares had previously diagnosed hyperlipidemia. The remaining mare had prolonged colitis as a comorbid condition.

The most common fetal presentation was cranial with an abnormal limb position in 27% (21/78), followed by neck flexion in 23% (18/78) and caudal presentation with bilateral hip flexion in 17% (13/78). In many cases, there was more than one instance of concurrent fetal malposition or malposture. The most common limb abnormalities were shoulder flexion in 48% (10/21) and carpal flexion in 14% (3/21). There were two reports in the current study of inadequate cervical dilation and two reports of fetopelvic disproportion.

Correction methods used in this group of miniature equids included CVD, AVD, cesarean delivery, and fetotomy. The most common correction method was CVD in 45% (35/78), followed by AVD in 35% (27/78), cesarean delivery in 17% (13/78), and fetotomy in 4% (3/78). No reported complications were clearly related to fetotomy. Four cuts were required in the one fetotomy case in which the number of cuts was recorded. In general, our practice follows an approach of allowing a combined 30 min for CVD and AVD before proceeding to a cesarean delivery; however, the decision was ultimately made by the attending clinician. In most cases, AVD and CVD were performed by Diplomates of the American College of Veterinary Emergency and Critical Care, but some cases of field dystocia were managed by non‐specialty‐boarded equine veterinarians.

Mares in this study were most commonly treated with flunixin meglumine (0.5–1.0 mg/kg, IV or PO, q 24 h or q 12 h), ceftiofur (2.2 mg/kg, IV, q 24 h), gentamicin (6.6 mg/kg, IV, q 24 h), or trimethoprim–sulfamethoxazole (30 mg/kg, PO, q 12 h). In addition to these treatments, 77% (60/78) of mares received intrauterine lavage regardless of whether they had RFM. Intrauterine lavage was performed both in the field or in hospital. The number of uterine lavages a mare received varied based on the character of uterine lavage fluid and the presence of clinical signs associated with metritis (pyrexia, malaise, vaginal discharge, etc.). Treatments were similar between mares that had cesarean delivery and other forms of delivery, with the exception of incision monitoring and care. All mares that required surgery were able to walk back from the recovery area to the ICU stall.

Of the hospitalized mares, 84% (56/67) received IV fluids, whether it was one or multiple fluid boluses or a continuous rate infusion (CRI). Of the mares receiving CRI fluids, 54% (30/56) also received a lidocaine CRI for at least 12 h. In general, our practice uses a lidocaine infusion rate of 0.05 mg/kg/min without a loading bolus. Partial parenteral nutrition was used in 42% (28/67) of hospitalized mares and was most commonly a dextrose CRI. The rate of dextrose CRI was variable, but the hospital's general practice is not to exceed 1 mg/kg/minute. Only two mares, both of which were critically ill (one with colitis, one with postoperative ileus), received combined amino acids and dextrose.

## 4 Discussion

This is the first report that focuses on a large number of dystocias in miniature equids. The survival rate for mares (94%) was good, but the survival rate for foals (17%) was poor. More than 80% of dystocias were corrected with either AVD or CVD, indicating that cesarean delivery in miniature equids is not commonly required for resolution of dystocia. Abnormal limb position and neck flexion are common causes of dystocia in miniature equids, but the caudal presentation with bilateral hip flexion position is also frequently encountered. RFM and metritis are common complications after dystocia in miniature equids.

Survival of mares was similar to previously reported values for full‐sized horses [4]. In our study population, there was little manipulation of the fetus by nonveterinarians or veterinarians on the farm. Early referral with few attempts at fetal manipulation may contribute to improved mare survival [2]. In small ruminants, increased attempts and duration of manipulation increase the risk of uterine trauma, which compromises doe survival [13]. Another potential factor contributing to better survival in mares may be close proximity to a referral center. The duration of dystocia can greatly increase the likelihood of a poor outcome for both the mare and the foal [8].

Foal survival was poor, similar to previous results in full‐sized horses [4]. Decreased foal survival could be related to fetal stress as a result of the dystocia. However, meconium staining, a hallmark of fetal hypoxia, was only reported in one case. It is also likely that many mares presenting with dystocia may have had foals that died in utero.

The frequency of cesarean deliveries reported in the current study in miniature equids with dystocia was similar to that reported for full‐sized horses (15%–25%) [2, 7, 11]. However, it was often unclear whether there were financial limitations that precluded cesarean delivery. In one study in small ruminants, 95% of animals presenting with dystocia required a cesarean delivery [14]. This discrepancy between frequencies of cesarean delivery in small ruminants and horses may be related to the cost of procedure. The most common reasons for surgery in small ruminants were inadequate cervical dilation, vaginal prolapse, fetopelvic disproportion, and pregnancy toxemia. Many of these conditions are less common in horses, which may explain the difference in the requirement for surgery.

Fetal malpositioning in full‐sized horses is commonly caused by abnormal limb or neck positioning, similar to that seen in the current study [3]. However, caudal presentation with bilateral hip flexion positioning was 17% in miniature equids in this study compared with another study reporting a rate of 4% in full‐sized horses [3]. Frazer et al. reported a greater prevalence of caudal presentation with bilateral hip flexion positioning, at 14% [5]. A better understanding of the factors leading to the caudal presentation with bilateral hip flexion positioning could help to determine whether miniature equids have a reason to be at higher risk.

In general, the size of miniature equids compared with full‐sized animals makes the management of dystocia different. Anesthetizing and lifting a miniature horse on the farm is much more practical than anesthetizing and lifting a full‐sized horse. Conversely, some veterinarians’ arms may be too large to safely palpate miniature horses. The combination of these factors may influence how dystocia is managed.

One limitation of the current study was the amount of missing data, which is common in retrospective studies. However, dystocia is a true emergency, and in many cases, presenting temperature and heart rate were not recorded. Similarly, other parts of the signalment may not have been confirmed. Long‐term follow‐up was also limited in many cases. Because of the nature of the referral practice, patients sometimes came to the facility on an emergency‐only basis but did not receive further ongoing care.

Another limitation of the study was that the duration of most of the cases of dystocia was not recorded. Many foalings were unobserved; therefore, the clients were unaware of the starting time of labor. In some cases, the treating veterinarian may have estimated the duration of dystocia, but an accurate time was often not available. Knowledge of the duration of dystocia would have been a key variable when evaluating foal survival. A future prospective study could attempt to better capture these missing data, but ultimately parturition start time is unknown in many cases of dystocia.

In conclusion, survival is good for miniature equid mares receiving emergency care for dystocia. Fetal malpositioning is similar to full‐sized horses, but there may be a higher prevalence of caudal presentation with bilateral hip flexion positioning. Dystocia in miniature equids can usually be corrected with CVD or AVD.

## Conflicts of Interest

The authors declare no conflicts of interest.

## Endnote

a GraphPad Prism version 8.2.1 for Windows, GraphPad Software, La Jolla, CA.
